# Optical Monitoring and Detection of Spinal Cord Ischemia

**DOI:** 10.1371/journal.pone.0083370

**Published:** 2013-12-16

**Authors:** Rickson C. Mesquita, Angela D’Souza, Thomas V. Bilfinger, Robert M. Galler, Asher Emanuel, Steven S. Schenkel, Arjun G. Yodh, Thomas F. Floyd

**Affiliations:** 1 Department of Physics and Astronomy, University of Pennsylvania, Philadelphia, Pennsylvania, United States of America; 2 Institute of Physics, University of Campinas, Campinas, São Paulo, Brazil; 3 Department of Anesthesiology, Stony Brook University Medical Center, Stony Brook, New York, United States of America; 4 Department of Biomedical Engineering, Stony Brook University Medical Center, Stony Brook, New York, United States of America; 5 Department of Surgery, Stony Brook University Medical Center, Stony Brook, New York, United States of America; 6 Department of Neurosurgery, Stony Brook University Medical Center, Stony Brook, New York, United States of America; Glasgow University, United Kingdom

## Abstract

Spinal cord ischemia can lead to paralysis or paraparesis, but if detected early it may be amenable to treatment. Current methods use evoked potentials for detection of spinal cord ischemia, a decades old technology whose warning signs are indirect and significantly delayed from the onset of ischemia. Here we introduce and demonstrate a prototype fiber optic device that directly measures spinal cord blood flow and oxygenation. This technical advance in neurological monitoring promises a new standard of care for detection of spinal cord ischemia and the opportunity for early intervention. We demonstrate the probe in an adult Dorset sheep model. Both open and percutaneous approaches were evaluated during pharmacologic, physiological, and mechanical interventions designed to induce variations in spinal cord blood flow and oxygenation. The induced variations were rapidly and reproducibly detected, demonstrating direct measurement of spinal cord ischemia in real-time. In the future, this form of hemodynamic spinal cord diagnosis could significantly improve monitoring and management in a broad range of patients, including those undergoing thoracic and abdominal aortic revascularization, spine stabilization procedures for scoliosis and trauma, spinal cord tumor resection, and those requiring management of spinal cord injury in intensive care settings.

## Introduction

Spine trauma from motor vehicle accidents, falls, violence, and sports activities, occurs at rates ~60 cases per million-individuals per year [1-3], and sixty-five percent of patients who present with spinal cord injury (SCI) are amenable to intervention [4]. Following trauma, early surgical intervention to preserve or restore spinal cord blood flow and oxygen delivery can help prevent secondary injuries [5-9]. Similarly, elective spine stabilization procedures for congenital and degenerative spine deformities and tumor resections are frequently performed, and many of these patients experience major neurological sequelae [10-14]. Finally, open repair or percutaneous stenting of descending thoracic aortic aneurysms and dissections are carried out relatively frequently [15], and a recent investigation found that ~28% of these patients developed spinal cord ischemia, which in turn led to paralysis or paraparesis [16]. 

Surprisingly, even though ischemia plays a prominent role in spinal cord injury, tools available to measure spinal cord blood flow and oxygenation are extremely limited. The ability to measure spinal cord blood flow with laser Doppler Flowmetry (LDF) has been demonstrated in both animal and human studies [17]. These devices, however, measure flow in a very limited tissue volume that is in close proximity to the probe tip; LDF sampling volumes are estimated at 0.3-0.5 mm^3^. Additionally, positioning of the rigid probe is troublesome, the probes are prone to fracture, and they cannot be left in place for an extended period of time. Noninvasive methods for spinal cord blood flow measurement have been investigated; they include single photon emission computed tomography [18] and MRI-based arterial spin labeling [19]. Indeed MRI and CT may become excellent tools for the measurement of spinal cord blood flow and can even be expected to have superior spatial sensitivity. However, intra-operative monitoring with both MRI and CT is simply not feasible and these tools do not permit continuous monitoring. 

Currently, the only methods employed for assessment of spinal cord ischemia are based on somatosensory (SSEP) and motor (MEP) evoked potentials. These technologies measure the integrity of posterior spinal somatosensory and anterior/lateral spinal motor tracts, respectively. When combined, they can identify injury, offer insight into the impact of particular interventions, and provide an opportunity to limit or reverse injury [20,21]. Even with MEP, alerts may be delayed relative to the inciting event by 10-20 minutes or more [22,23] because signal transmission failure occurs when axons begin to die. Such a delay diminishes the opportunity to rescue threatened tissues. Neuro-electrophysiological monitoring can also be adversely influenced by anesthetic management [24,25], hypothermia [26], limb ischemia, and technological malfunctions (e.g., lead displacement, disconnection, etc.). “False negatives,” wherein patients have awakened with serious deficits in spite of normal evoked potentials, and “false positives,” wherein patients have awakened without deficits in spite of loss or degradation of signal, have both been reported with SSEP [27] and MEP [28,29] monitoring. Finally, accurate and timely interpretation of these data requires the continuous presence of a skilled physician with expertise in neuro-electrophysiological monitoring; such data are not easily interpretable by anesthesiologists, surgeons, neurointensivists, or nurses. Despite these limitations, MEP and SSEP remain the “gold standard” for functional monitoring of the spinal cord during aortic, spine, and spinal cord surgery.

Spinal cord ischemia, either as the inciting event, or via secondary spinal cord ischemia after trauma, contributes importantly to the outcomes of paralysis and paraparesis. The obstacles to overcome for prevention and treatment of spinal cord injury are daunting, and achieving these goals is likely to occur incrementally. Clearly, in order to prevent or ameliorate neurological injury due to spinal cord ischemia, it is desirable to detect it at its earliest time-point and follow its progress. To this end, we introduce and demonstrate a new optical tool that permits direct monitoring of spinal cord blood flow and oxygenation. Indeed, because blood flow and oxygenation maintenance and reinstitution are at the heart of spinal cord integrity and recovery, such a device holds promise to have substantial clinical impact, filling a critical void in current spinal cord monitoring technology and offering a new approach to detect and assess treatment of spinal cord ischemia. Herein we report our initial experience in testing the novel fiber optic monitor for the detection of spinal cord blood flow and oxygenation. The results suggest that the new instrument has untapped value for clinical procedures such as aortic revascularization, spine stabilization for trauma, surgical correction of spine deformities, and resection of spinal cord tumors. In addition to surgical applications, the probe could facilitate management of spinal cord ischemia in the ICU and could aid in preoperative prediction of the risk for spinal cord ischemia during aortic surgery. Finally, the demonstrated potential of this simple hemodynamic spinal cord monitoring tool should facilitate targeted laboratory investigations into the efficacy of new therapeutic approaches aiming to prevent or ameliorate spinal cord ischemia. 

## Materials and Methods

### Ethics Statement

All animal studies were approved by the Institutional Animal Care and Use Committee (IACUC) of the Stony Brook University Medical Center, where the experiments were carried out. Animal facilities are accredited by the American Association for the Accreditation of Laboratory Animal Care.

### Animals and experimental protocol

The sheep and human spines are close in bony dimensions, to include spinal canal depth and width, especially in the thoracic and lumbar spines [30]. For both species, canal width is greater than depth, thus producing a typically oval shape that is most pronounced in the lumbar region. The human spinal canal is wider and deeper in the antero-posterior plane than sheep [31], potentially inferring that safety demonstrated in the sheep model should predict the same, if not greater safety profiles in a human. There are numerous reports of the use of the sheep model to study spinal cord injury. Kaplan [32], Moomiaie [33], and Bockler [34] describe the use of the sheep model for detecting interruption of spinal cord blood flow during aortic cross-clamping. Numerous reports exist for the testing of the epidural approach for anesthesia and for attempts at protecting against spinal ischemia in sheep [35-39]. In the sheep, the vertebral processes can be more easily palpated than in the pig, another model used in spinal cord research.

We therefore employed ten adult Dorset sheep in the studies reported. The animals were approximately 2 years old, and weighed approximately 30-40 kg. Animals were pre-treated with glycopyrrolate (0.02 mg/kg, IM). Anesthesia was induced with ketamine (10-20 mg/kg, IM), and animals were intubated and anesthesia maintained with isoflurane (1.5-3.0%) via controlled ventilation. The paralytic agent vecuronium (0.1 mg/kg, IV) was employed after obtaining a deep plane of anesthesia. These agents were used to prevent movement during electrocautery, during opening the chest, or during laminectomy, as movement of the animal due to muscle or nerve stimulation may result in injury to the heart or spinal cord during dissection. Animals were anesthetized and monitored under the supervision of the Department of Laboratory Animal Resouces veterinarian.

### Surgical and Monitoring Procedures

After adequate general anesthesia was established, the sheep was positioned prone.  When relevant, intra-operative fluoroscopy was used to localize the appropriate levels of surgery.  Otherwise, an estimation based on palpation was used to allow for access to the thoracolumbar junction.  All surgical procedures were undertaken personally by the attending neurosurgeon.  High powered loupe magnification and headlights were used.  A midline incision was made with a #10 scalpel blade down to the spinous processes.  A self-retaining retractor was then placed. Using a Bovie monopolar electro cautery, a subperiosteal dissection was performed to expose the spinous processes, lamina and medial facets.  A deeper retractor was then placed to hold the muscle out of the field. A Leksell rongeur was then used to remove the intra-spinous ligaments as well as the spinous processes themselves. The rongeur as well as a burr was used to thin the lamina further. A currette and Penfield dissector was then used to delineate the interlaminar space. A thin footed Kerrison was used to carefully remove the lamina creating a trough that was sequentially widened until the dura of the spinal cord was well seen.  Depending on the needs of the experiment, one to four levels were decompressed and exposed. When indicated, the dura was opened with the tip of an #11 scalpel blade allowing for passage of the detector.

Femoral and carotid arterial and venous cannulae were placed after anesthetic induction, for the measurement of mean arterial pressure and microsphere sampling. A left thoracotomy was performed via the T6-10 interspace allowing for left atrial cannulation for the injection of fluorescent microspheres. An intra-aortic balloon was placed via the left femoral artery and advanced to a level immediately below the common cephalic trunk. The fiber optic probe was placed via either open or percutaneously via a 16 gauge Tuohy needle in the lumbar interspace, with the epidural space identified by loss of resistance to air or saline, and the intrathecal space identified by the presence of cerebrospinal fluid (percutaneous). In the instance of a laminectomy, the highly flexible probe was placed using loupe magnification and was advanced superiorly or inferiorly under intact laminae to lay within the epidural or intrathecal space. Ventilatory rate and volume, systemic oxyhemoglobin saturation (pulse oximetry), and mean arterial pressure were continuously monitored (PowerLab, ADInstruments, CO). All animals were euthanized under general anesthesia at experiment end.

In all sheep, multiple pharmacological, physiological, and mechanical interventions were performed after baseline measurements. Hypertension was induced via boluses of phenylephrine (400 μg) or vasopressin (4 units). Hypotension was induced with nitroprusside (400 μg). Systemic hypoxia and hypercarbia were created via respiratory arrest. Spinal cord ischemia was induced via inflation of the intra-aortic balloon (Koda Cook, Bloomington, IL) to occlude the aorta. Occlusion was confirmed radiographically, via direct visual inspection through the open chest, and via loss of femoral arterial pressure. In all experiments, mean arterial pressure and spinal cord blood flow were allowed to return to baseline prior to the subsequent intervention.

### Diffuse Optical measurements

**Figure 1:**
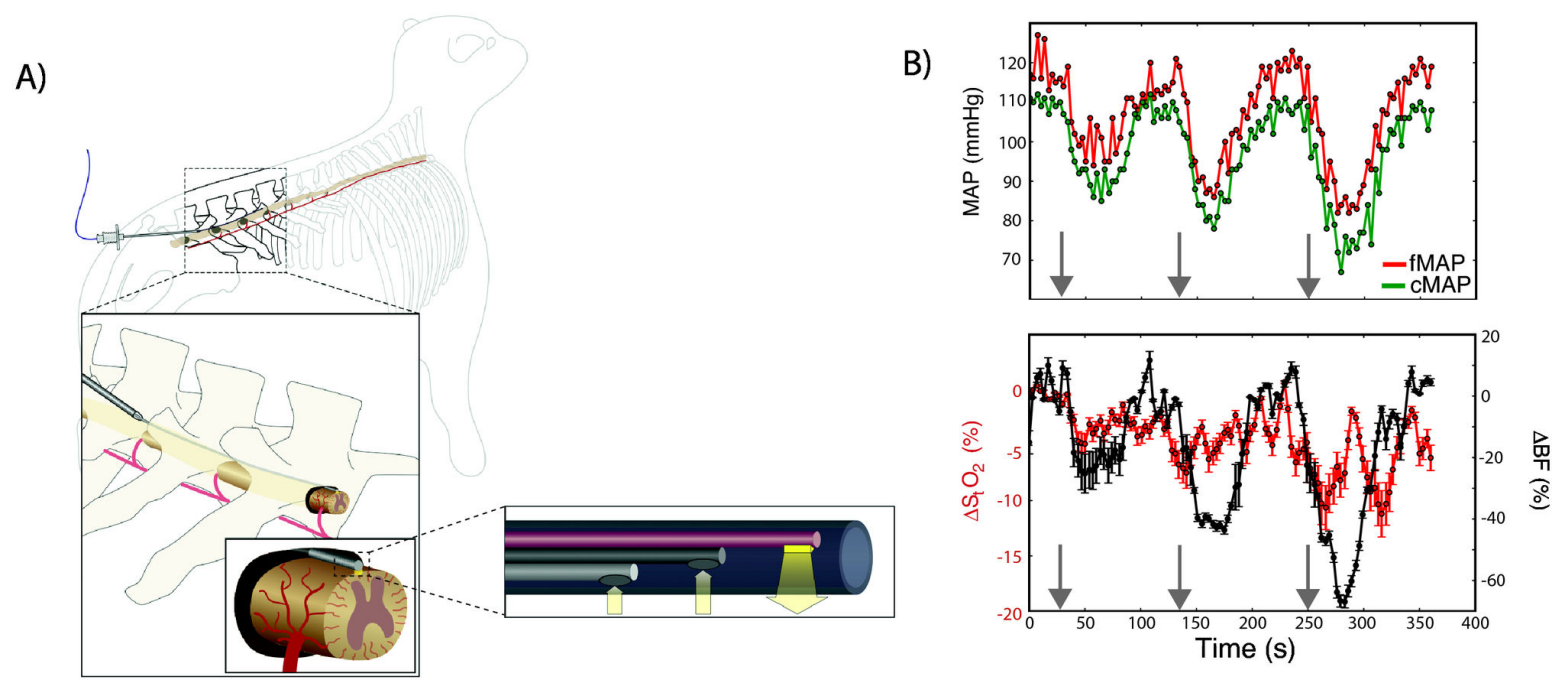
Fiber optic instrument validation. (A) Detailed schematics of the experiment, featuring the instrument and its thin fiber optic probe. (B) In this experiment the instrument detected consistent physiologic changes due to three consecutive boluses of nitroprusside (gray arrows); the first bolus was 200 μg and the subsequent ones were 400 μg. Arrows indicate the time when the drug was administered. Each time point represents the mean value estimated by averaging the data from the two source-detector separations; the error bars represent the minimum and maximum blood flow/oxygenation changes measured across the source-detector separations. In the oxygen saturation time-course, the error bars also account for the uncertainty in the DPF assumed to estimate hemoglobin concentration changes. (MAP = mean arterial pressure, femoral (f) or carotid (c); ΔBF = changes in blood flow; ΔS_t_O_2_ = changes in tissue oxygen saturation).

The optical equipment was a homemade instrument that employs diffuse optical (DOS) and correlation (DCS) spectroscopies [40,41]. The DOS module employs one source fiber switched between three amplitude modulated (70 MHz) laser diodes in the near-infrared (686, 785 and 830 nm). The switching time was adjusted to be 150 milliseconds. Two photomultiplier tubes collected modulated light emerging from the spinal cord. The DCS module used a continuous-wave, long-coherence length (>20 m), 785 nm laser. Two photon-counting avalanche photodiodes collected DCS light and were fed to a four-channel autocorrelator board that computed the temporal intensity autocorrelation function of the collected light. Optical fibers deliver (collect) light to (from) the spinal cord. The optical probe was a custom made long and thin specifically for the purpose of this study. It is 3 m long so that the instrument can be positioned distant from the procedure, and its diameter is a little less than 1 mm (see Figure 1A for details on the probe). The optical probe is made of glass fibers covered by Teflon, and employed two source-detector separations at 1 and 2 cm. The whole acquisition cycle takes approximately 3.5 seconds (3 seconds for DCS and approximately 0.5 seconds for DOS).

The transmitted light intensity variation for each source-detector pair at each wavelength was processed to derive chromophore concentration changes, i.e., oxy- (ΔHbO_2_) and deoxy-hemoglobin (ΔHb) concentration changes, using the modified Beer-Lambert law with a differential path-length factor (DPF) of 4 for all three wavelengths [40]. The DPF value was chosen based on our previous experience with other tissues, since no values are available for spinal cord in the literature. From the oxy- and deoxy-hemoglobin concentration changes, we determined changes in tissue oxygen saturation, (ΔS_t_O_2_), relative to a baseline period. Tissue oxygen saturation, S_t_O_2_(t), is defined as the ratio, HbO_2_(t)/[HbO_2_(t) + Hb(t)], where for each chromophore, x(t) = x(t_0_) + Δx; here, t_0_ denotes the baseline period, the averages during baseline are taken over a period of two minutes before the interventions, and x = HbO_2_, Hb, respectively. In order to determine absolute baseline hemoglobin concentrations, we employed a white-light spectrometer system independently. Details about the instrument and data analysis can be found elsewhere [42-45]. Briefly, our white-light spectrometer utilizes the same principles of diffuse optics as DOS, but its spectral content enables experimenters to derive absolute hemoglobin concentrations at a single point in time (i.e., in contrast to the DOS system which provides continuous measurement of the relative changes in hemoglobin concentration). Across six trials with the white-light spectrometer, the mean (standard deviation) absolute concentrations for HbO_2_ and Hb were 27.2 (7.8) μmol and 10.5 (1.7) μmol, respectively. We used these values as our estimate of baseline absolute hemoglobin concentrations in subsequent analyses.

Measures of spinal cord blood flow were estimated from DCS data by fitting the measured intensity autocorrelation function to the solution of the photon correlation diffusion equation in the semi-infinite geometry with extrapolated zero boundary conditions [41,46]. The Brownian motion model was used to approximate the mean-square particle displacement of the moving scatterers in the tissue, and thus to derive a spinal cord blood flow index (BFI) [41]. Relative changes in blood flow (ΔBF) were calculated from ΔBF(t) = ΔBFI(t) = BFI(t)/BFI(t_0_) – 1, where t_0_ denotes the baseline period and baseline data are derived from two-minute averages of BFI before the interventions.

For each time point, we averaged ΔBF and ΔS_t_O_2_ from the two source-detector separations. This procedure enabled us to estimate a blood flow/oxygenation time-series during each experiment. In the figures, each time point thus represents the mean value estimated from the two source-detector separations, and the error bars represent the corresponding minimum and maximum blood flow/oxygenation changes. In addition, for oxygenation changes, the choice of a DPF value of 4 may lead to systematic errors in quantification; therefore, we analyzed the effect of varying DPF from 2 to 8 on our data, and this effect was propagated and accounted for in the reported error bars.

### Microsphere measurements

Microspheres were employed to validate DCS flow measurements. Multi-colored, nonradioactive stable-isotope labeled microspheres of 15-μm diameter were injected into the left atrium (1 ml, 2x10^6^ microspheres/ml, STERIspheres, Biopal, MA). Differently colored microspheres were used to perform measurements at different time points. Following euthanasia, the spinal cord was labeled, resected, and preserved. Spinal cord tissue blocks were then prepared for subsequent analysis of relative microsphere concentrations [47-50]. 

### Statistical Analysis

The changes due to intervention were calculated from the highest/lowest blood flow/oxygenation value in the time-course during the intervention period. For each intervention, data were summarized using the mean change and standard error, i.e., by averaging over all trials and all animals. Non-parametric Wilcoxon signed rank tests were used to assess whether the observed differences were statistically distinct from those at baseline. Similarly, Wilcoxon rank sum tests were used to compare hemodynamic changes measured with the different surgical approaches (subdural, epidural, percutaneous). 

The comparison between spinal cord blood flow measured by DCS and the microspheres techniques was carried out by correlating the simultaneous point measures across all animals and trials. The degree of correlation was measured by the Pearson correlation coefficient. The ratio of DCS changes to microsphere changes was obtained from the slope of the curve. All data analyses and statistics were performed with Matlab (MathWorks Inc., Natick, MA).

## Results

Measurements with the fiber optic device are feasible and sensitive. The fiber optic instrument is shown schematically in Figure 1A. It utilizes the principles of diffuse optics to probe the vasculature of thick tissues surrounding the spinal cord. Diffuse optical spectroscopy (DOS) is employed to measure changes in tissue hemoglobin concentration, providing a direct measurement of tissue oxygen saturation changes (ΔS_t_O_2_) [40]. Diffuse correlation spectroscopy (DCS) is used to measure changes in tissue blood flow (ΔBF) [41]. Both methods utilize near infrared light because deep tissue penetration is required. The instrument was tested in ten adult Dorset sheep after placement in the mid- and lower-thoracic spinal regions.

To test the sensitivity of the DCS/DOS fiber optic probe we first examined its response to pharmacological and physiological perturbations with known, predictable responses. A representative example is shown in Figure 1B, wherein repeated episodes of hypotension elicited with nitroprusside boluses produced the expected decrease in spinal cord blood flow and modest decreases in oxygenation (Figure 1B). The induced hemodynamic variations were rapidly and reproducibly detected by the optical device. In total, the optical probe successfully detected significant decreases (p = 0.004) in spinal cord blood flow associated with the bolus of hypotensive agents in 9/9 trials, and significant increases (p = 0.003) in spinal cord blood flow associated with the bolus of hypertensive agents in 12/12 trials. Across all animals, the mean (standard error) change in blood flow associated with the hypotensive and hypertensive challenges was, respectively, −38.9 (3.6) % and 52.8 (3.8) %. The minimum (maximum) flow change measured by the device during the hypotensive and hypertensive challenges was, respectively, −25 % (−55 %) and 40 % (70 %). The spinal cord blood flow, measured for the first time by optics, closely paralleled changes in femoral and/or carotid mean arterial pressures (fMAP and cMAP, respectively) during hypotensive and hypertensive challenges. On multiple occasions, blood flow returned to baseline before MAP returned to baseline, an effect that may document pressure autoregulation of blood flow by the spinal cord. Finally, systemic hypoxia and hypercarbia, induced by breath-hold, resulted in an increase in spinal cord blood flow and fall in oxygenation, all consistent with physiologic expectations. In three animals, we measured a mean (minimum, maximum) increase of 93.3 (90, 100) % in blood flow, and a −13.7 (−15, −13) % decrease in oxygen saturation due to 5 minutes of breath-holding designed to elicit systemic hypoxia and hypercarbia; femoral and carotid mean arterial pressures were unaffected in this smaller study.

A second group of measurements demonstrated that the optical instrument can be employed in either epidural or subdural positions, and can be placed via laminectomy or via a percutaneous approach. Briefly, the probe was positioned: 1) upon the posterior cord after thoracic laminectomy, within the subdural space; 2) upon the posterior cord after thoracic laminectomy, within the epidural space (both locations were confirmed under direct vision), and finally, 3) upon the posterior cord after percutaneous placement, through a 16 gauge Tuohy needle positioned at the lumbar spine, and after cephalad advancement. Specifically, the feasibility for employing the optical probe in both epidural and subdural positions (approaches 1 and 2 described above) was tested, along with the ability to place the probe via laminectomy (Movie S1) or a percutaneous approach (Movie S2). The percutaneous approach, in particular, is similar to current techniques used to place spinal drains and epidural catheters. 

**Figure 2:**
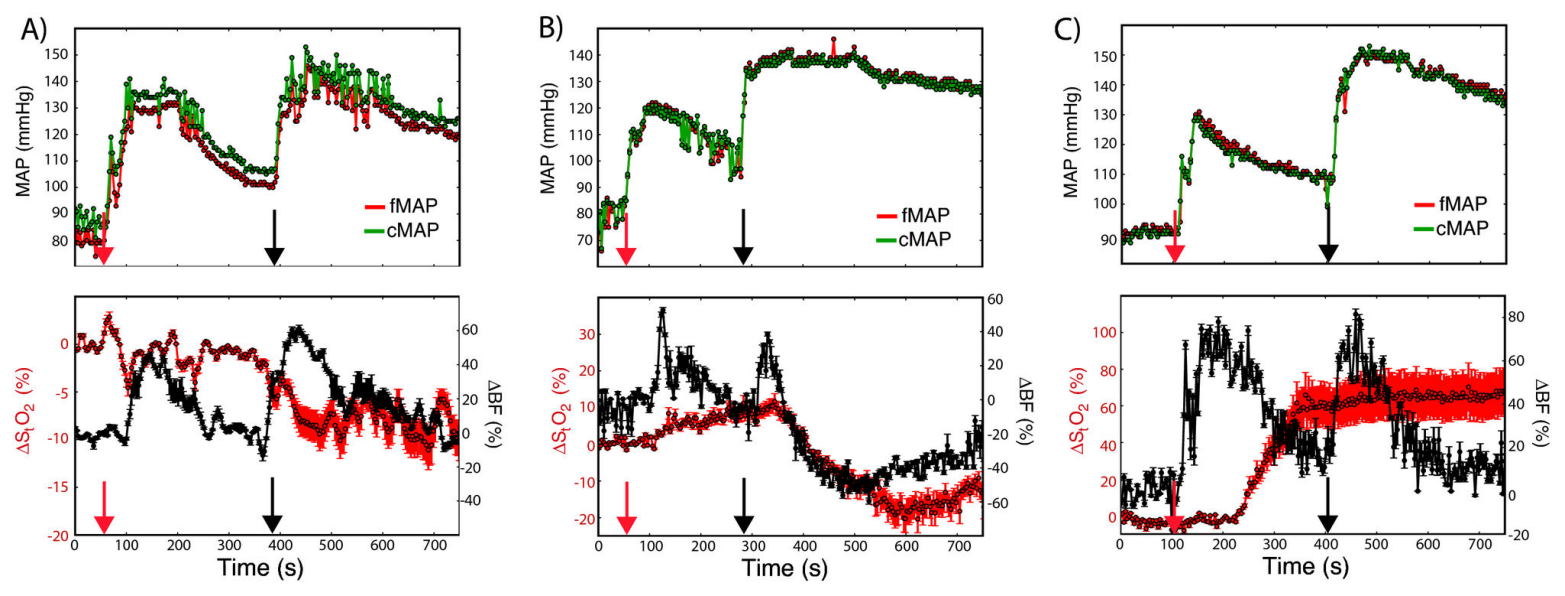
Hemodynamic changes measured through different approaches. Mean arterial pressure and hemodynamic changes measured during injection of boluses of phenylephrine injection (red arrow) followed by vasopressin injection (black arrow) in (A) subdural, (B) epidural, and (C) subdural positions after percutaneous approach. Arrows indicate the instant the drug was administered. Each time point represents the mean value estimated by averaging the data from the two source-detector separations; the error bars represent the minimum and maximum blood flow/oxygenation changes measured across the source-detector separations. In the oxygen saturation time-course, the error bars also account for the uncertainty in the DPF assumed to estimate hemoglobin concentration changes. (MAP = mean arterial pressure, femoral (f) or carotid (c); ΔBF = changes in blood flow; ΔS_t_O_2_ = changes in tissue oxygen saturation).

In all positions, and using both open and percutaneous approaches, hemodynamic changes in the spinal cord due to pharmacological, physiological, and mechanical interventions were measured. Hypertensive challenges, for example, produced expected increments in spinal cord blood flow and modest oxygenation variation (Figure 2). Importantly, spinal cord blood flow measured at the epidural position was not statistically different from spinal cord blood flow measured at the subdural position for all different interventions (p = 0.87). Measurements in both positions provided nearly identical perfusion information, thereby demonstrating that the presence of an intervening dural layer did not impair detection of vascular dynamics. Finally, tissue flow measurements after percutaneous positioning were consistent with those obtained when the probe was placed under direct vision. Percutaneous positioning of the probe yielded hemodynamic changes that were not statistically different from changes measured when the probe was placed under direct vision (p = 0.65), an observation of substantial practical import, since the percutaneous placement of similar epidural catheters and cerebrospinal fluid drains has demonstrated an excellent safety profile, over many years in clinical practice.

**Figure 3:**
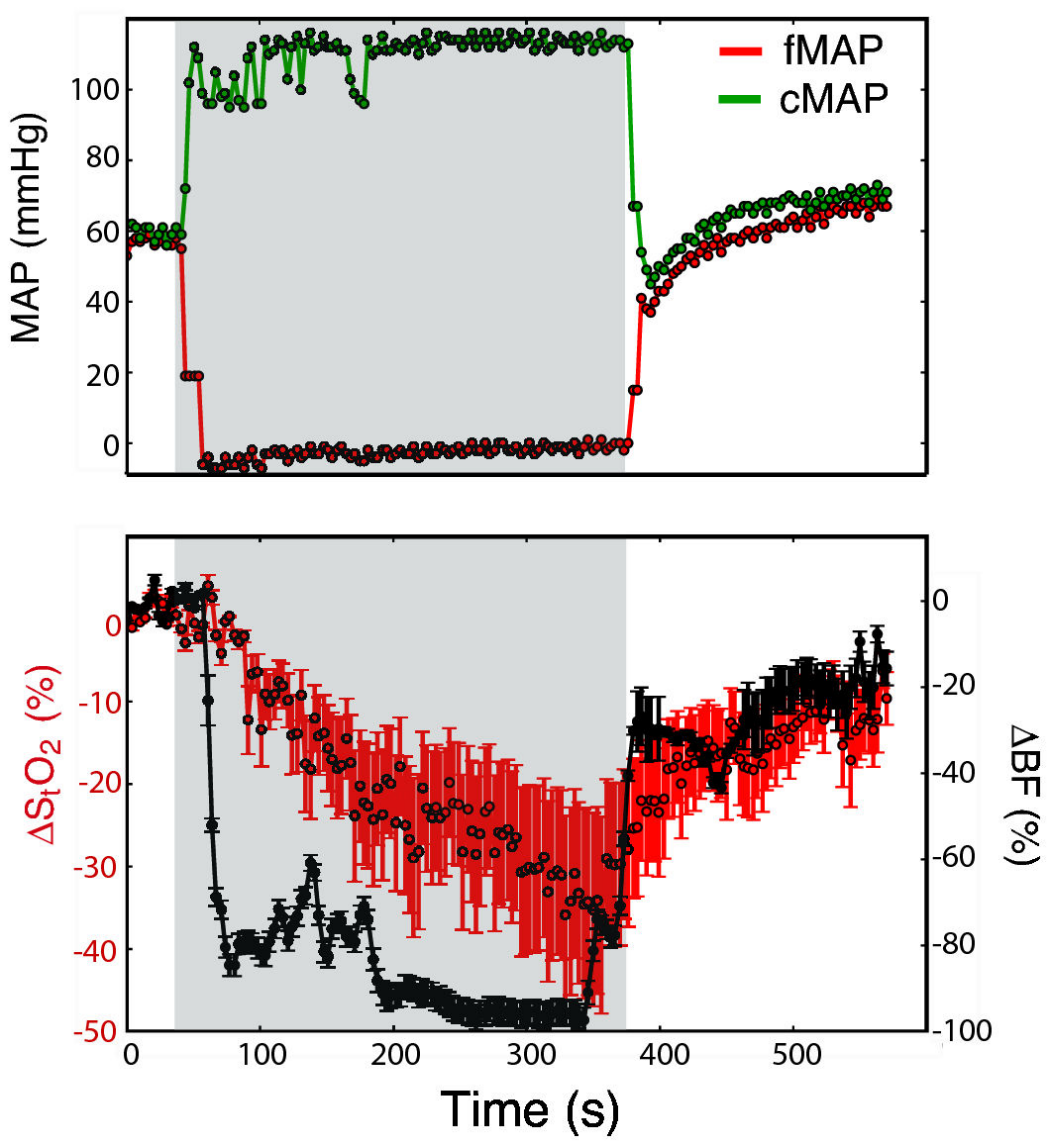
Hemodynamic monitoring during ischemia. Blood flow (ΔBF), oxygen saturation (ΔS_t_O_2_) and mean arterial pressure (MAP, femoral (f) or carotid (c)) changes measured percutaneously during intra-aortic balloon inflation (grey area).

A third group of measurements showed that the optical instrument can sensitively and rapidly detect spinal cord hemodynamic changes due to aortic occlusion. We tested the sensitivity of the probe for detecting blood flow and oxygenation changes by using an ischemia model that replicates what is seen during thoracic aortic surgery, wherein the intercostal blood supply to the spinal cord from the aorta is interrupted via clamping, resection, or placement of an endoluminal graft. As previously described in the Surgical and Monitoring Procedures subsection, the aorta was occluded in our sheep model via inflation of an endo-aortic balloon just below the common cephalic trunk. Occlusion was maintained for 5 minutes. Immediately following aortic occlusion, spinal cord blood flow was observed to fall precipitously to levels 90% below baseline. During the period of occlusion, oxygenation fell more slowly, decreasing 20-40% below baseline (Figure 3). This pattern was detected in 14/14 aortic occlusions, both in open and percutaneous DCS/DOS fiber optic probe placement approaches. Across all trials and all animals, we observed a significant decrease (mean (standard error)) in spinal cord blood flow and oxygenation of −97.5 (12.8) % (p = 0.002) and −23.1 (4.9) % (p = 0.016), respectively. 

The final experiment compared optical changes with independent measurements employing multi-colored microspheres for the assessment of flow at baseline, and following aortic occlusion and hypertensive interventions (N = 8). Spinal cord sections from immediately superior and inferior to the position where fiber optic probe was placed were collected after euthanasia for microsphere quantification [47]. A direct comparison between the flow index of the optical device and the microsphere technique is hard to achieve, because the latter reflects perfusion resulting from several cardiac ejections, only offering a “snapshot” of flow, while the fiber optic probe measures flow continuously. Nevertheless, across all 8 data points collected, the techniques are highly correlated (R = 0.93), with slope close to unity (i.e., DCS/microspheres = (0.90 ± 0.16)). Such a strong linear relationship suggests that the optical device is indeed measuring spinal cord blood flow. Furthermore, the fact that the probe detected a marked loss of flow during aortic occlusion that correlated tightly with a near complete loss of blood flow as validated by microspheres would strongly suggest that the device was indeed detecting a loss of spinal cord blood flow, the magnitude of which would cause ischemia.

## Discussion

Spinal cord injuries occur at high rates worldwide and represent a high cost for the society and the individual. In cases where patients are amenable to intervention, detection and monitoring of spinal cord hemodynamics is crucial for avoiding secondary ischemia in the intensive care unit or intraoperative setting. Failure to detect spinal cord ischemia may result in permanent paraplegia, and early detection of spinal cord ischemia enables implementation of therapeutic interventions that can stabilize or reverse injury, and decrease morbidity. Unfortunately, no technology is available to directly and continuously monitor the impact of interventions on spinal cord blood flow and oxygenation currently.

In this study, we introduce and demonstrate a fiber optic technology based on diffuse optical principles, that enables direct, quantitative, and continuous monitoring of spinal cord blood flow and oxygenation. The instrument was tested in adult Dorset sheep after placement in the mid- and lower-thoracic spinal regions, using both open (laminectomy) and closed (percutaneous) insertion techniques. The device proved capable of measuring spinal cord blood flow and oxygenation when placed immediately adjacent to the spinal cord within the intrathecal, above the dura in the epidural space (open approaches), and via percutaneous approach.

We tested the fiber optic probe’s response to pharmacologic, physiological, and mechanical interventions, designed to alter mean arterial pressure, induce hypoxia and hypercarbia, and to elicit spinal cord ischemia. The induced variations of spinal cord blood flow and oxygenation were rapidly and reproducibly detected by the diffuse optical device. Hemodynamic changes in the spinal cord were consistent with expectations in all cases. Lastly, variations in spinal cord blood flow measured by the fiber optic probe were in tight agreement with concurrent measurements of flow made with multi-colored microspheres, the “gold standard” for measurement of blood flow in the laboratory. While the high correlation is promising, more validation data points at different interventions are needed to derive a more accurate quantitative change in blood flow by DCS. Most of the simultaneous microspheres/DCS data were collected during ischemia interventions due to the potential for the technique to detect ischemia, which might introduce a bias in the estimated slope of DCS/microspheres validation. More simultaneous measurements of DCS and microspheres during different interventions that elicit different magnitudes of flow changes will be performed in the near future.

Regarding safety, the device can be placed via both open and percutaneous approaches using methods currently in clinical use and with proven safety [51,52]. Complications associated with instrumentation of the spine could include principally hematoma, infection (arachnoiditis, meningitis, and abscess), direct nerve injury, headache, cerebrospinal fluid leak, and intracranial hemorrhage. Hazards associated with fiber optic technology also include the potential for thermal injury, electrical shock, and toxicity related to materials [53]. Our probe is coated with Teflon (PTFE), a thermoplastic polymer used to create the flexible, heat-shrink tubing enclosing the fiber-optic probe. Teflon has been widely used in medical applications such as catheters and vascular grafts. It is resistant to high temperatures of up to 230°C. Its biocompatibility has been classified as USP-VI and extensive biocompatibility testing is not required.

Together, the results presented in this study indicate that the novel optical instrumentation presents a promising methodology for probing spinal cord hemodynamics non-invasively and continuously. A few steps are still required, however, before moving the technique to clinical applications in the near future. Technologically, improved real-time data processing and display will represent an important step toward unattended bedside monitoring in the neuro-ICU. A full biocompatibility analysis of the probe remains to be undertaken. From a methodologic perspective, the ability to separate between the anterior and posterior circulation hemodynamics and correlation with both motor and somatosensory evoked potential monitoring will permit discrimination of threats to the motor or somatosensory tracts. Clinically, the tolerance of the spinal cord to decrements in blood flow is currently not known, and the device introduced in this manuscript presents a pathway to produce further research about this topic. Ultimately, correlation of spinal cord blood flow changes with neurological deficits will be important to understand.

In summary, the device we have introduced and demonstrated has the potential to fill an important gap in neuromonitoring for the prevention and amelioration of spinal cord ischemia. It is particularly attractive because it enables direct, continuous measurement of spinal cord blood flow and oxygenation and immediate detection of changes in these parameters, with high fidelity.

## Supporting Information

Movie S1 **Probe positioning during subdural measurement.** The movie shows how the fiber optic device is placed through durotomy over the spinal cord.
(M4V)

Movie S2 **Probe positioning during percutaneous measurement.** The movie shows how the fiber optic device is placed percutaneously via a 16 gauge Tuohy needle. It is possible to see the probe flashing over spinal cord at laminectomy site.
(M4V)
